# Selected Livestock-Associated Zoonoses as a Growing Challenge for Public Health

**DOI:** 10.3390/idr14010008

**Published:** 2022-01-13

**Authors:** Kacper Libera, Kacper Konieczny, Julia Grabska, Wiktoria Szopka, Agata Augustyniak, Małgorzata Pomorska-Mól

**Affiliations:** 1 Department of Preclinical Sciences and Infectious Diseases, Poznan University of Life Sciences, Wołyńska 35, 60-637 Poznań, Poland; kacper.libera@up.poznan.pl (K.L.); agata.augustyniak@up.poznan.pl (A.A.); 2 Department of Internal Diseases and Diagnostics, Poznan University of Life Sciences, Wołyńska 35, 60-637 Poznań, Poland; kacper.konieczny@up.poznan.pl; 3 Faculty of Veterinary Medicine and Animal Science, Poznan University of Life Sciences, Wołyńska 35, 60-637 Poznań, Poland; julsgrabska@gmail.com (J.G.); wiktoriaszopka@gmail.com (W.S.)

**Keywords:** One Health, zoonotic pathogens, foodborne diseases

## Abstract

The aim of this paper is to review the most significant livestock-associated zoonoses. Human and animal health are intimately connected. This idea has been known for more than a century but now it has gained special importance because of the increasing threat from zoonoses. Zoonosis is defined as any infection naturally transmissible from vertebrate animals to humans. As the frequency and prevalence of zoonotic diseases increase worldwide, they become a real threat to public health. In addition, many of the newly discovered diseases have a zoonotic origin. Due to globalization and urbanization, some of these diseases have already spread all over the world, caused by the international flow of goods, people, and animals. However, special attention should be paid to farm animals since, apart from the direct contact, humans consume their products, such as meat, eggs, and milk. Therefore, zoonoses such as salmonellosis, campylobacteriosis, tuberculosis, swine and avian influenza, Q fever, brucellosis, STEC infections, and listeriosis are crucial for both veterinary and human medicine. Consequently, in the suspicion of any zoonoses outbreak, the medical and veterinary services should closely cooperate to protect the public health.

## 1. Background

**Figure 1:**
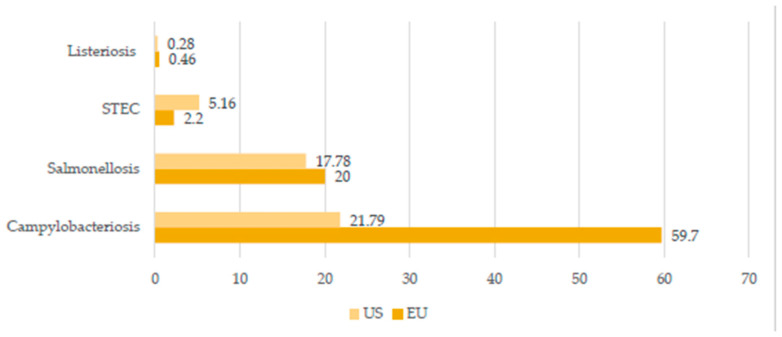
Selected livestock-associated zoonoses: comparison of the number of cases in United States (US) and European Union (EU) in 2019 (cases per 100,000 population). Created based on data from [5,7].

The twenty-first century is the age of globalization and urbanization, and is characterized by more and more free flows of people, animals, and goods around the world. Therefore, the conception of One Health gains importance like never before. The main assumption behind this idea is that the environment and human as well as animal health are intimately connected and interdependent. Any infection naturally transmissible from vertebrate animals to humans is called zoonosis. The pathogen transmission from animal to human is not only associated with the direct contact but also may occur via vectors or consuming animal products such as milk, meat, or eggs (foodborne diseases). Zoonotic diseases, particularly those associated with livestock and poultry, are becoming an increasing threat for public health due to different reasons. For example, the predictions suggest that the global human population will constantly increase and reach almost 10 billion by 2050 [1]. Consequently, it will result in a higher food demand. Thus, the livestock population is also expected to increase in order to cover the need for food, in particular regarding the high nutritional value of, for example, dairy or meat products. In 2020, the world meat and milk production was estimated at 337.2 and 906 million tonnes, respectively [2]. However, according to FAO, more than 70% of additional animal protein will be needed to feed the world by 2050, which suggests that animal production worldwide will be expected to grow. In turn, this potentially increases the risk of transmitting pathogens from animals to humans. The World Organization for Animal Health (OIE) suggests that 60% of pathogens that cause human diseases originate from domestic animals or wildlife [3]. Another fact is that 75% of emerging human pathogens are of animal origin [3]. For example, it has been suspected from the beginning that the original outbreak of SARS-CoV-2 was of zoonotic origin, possibly linked to a market in Wuhan, which sold a variety of animals including wild birds, poultry, fish, shellfish, and other exotic species [4]. It is important to note that the significance of particular zoonotic diseases differs within the continent and/or country mainly due to different zoohygienic conditions, human dietary habits, dominant livestock species, and legal environments. For example, according to the European Food Safety Authority (EFSA), the most frequent zoonoses in 2020 in the EU were campylobacteriosis, salmonellosis, Shiga-toxic Escherichia coli (STEC) infections, yersiniosis, listeriosis, and tularaemia, each reaching more than 1000 human cases requiring hospitalization [5]. Meanwhile, the Centre for Diseases Control (CDC) suggests prioritizing in the USA the following diseases and pathogens with zoonotic properties: influenza (zoonotic influenza A viruses), Salmonellosis (*Salmonella* species), West Nile virus, Plague (*Yersinia pestis*), emerging coronaviruses (*Coronaviridae*; i.e., severe acute respiratory syndrome (SARS-CoV) and Middle East respiratory syndrome (MERS-CoV)), Rabies (*Rhabdoviridae*, Lyssavirus), Brucellosis (*Brucella* species), and Lyme disease (*Borrelia burgdorferi*) [6]. A brief comparison of the chosen zoonoses incidences in the UE and US in 2019 is given in Figure 1 [5,7]. On the other hand, research from East Africa [8] revealed that the greatest concern regarding zoonoses is reserved for trypanosomiasis and brucellosis in this part of the world. From the Chinese point of view, major emerging zoonoses include SARS, Highly Pathogenic Avian Influenza (HPAI), rabies, Japanese encephalitis, brucellosis, and schistosomiasis japonica [9]. Therefore, this review aims to describe the most significant zoonotic diseases worldwide considering different farm animal species. A brief summary of zoonotic threats from livestock is given in Table 1. It is important to note that decent knowledge of these diseases and their transmission is crucial since it enables people to take action, including introducing proper risk assessment models. It involves the application of new technologies such as metagenomics, which is now the main method used to identify novel viruses and thus plays a central role in studies aimed at assessing zoonotic risk [10]. From a global point of view, the key reference regarding risk assessment models is the tripartite guide addressing zoonotic diseases, which was developed by the experts from the Food and Agriculture Organization of the United Nations (FAO), World Health Organization (WHO), and World Organization for Animal Health (OIE). Besides, constant epidemiological surveillance and report systems must be timely and efficient since surveillance in animals and humans is critical for the early identification and possible prediction of future outbreaks, allowing for preemptive action [11]. Similarly, timely, accurate, and reliable laboratory tests are critical for identifying etiologies of disease and to monitor both endemic and emerging zoonotic diseases in humans and livestock, which allows for implementing proper prevention as well as detection and response strategies [11]. However, it is important to note that animals other than livestock can also be a risk for human health including, e.g., dogs and cats (rabies), as well as wildlife (rabies, tularemia, and Lyme disease) [12,13]. Another important perspective regards climate change and the possible detrimental influence on vector-borne diseases, which may in the future expand and/or alter the geographical ranges of biological vectors and consequently the zoonotic diseases transmitted by them [14].

## 2. Q Fever

Q fever is a severe, zoonotic worldwide disease caused by *Coxiella burnetii*. This disease was first described by Derrick in 1937 following an epidemic fever outbreak among employees at a slaughterhouse in Brisbane (Australia) [26]. *Coxiella burnetii* is an obligate intracellular bacterium. Its cell wall is similar to that of Gram-negative bacteria but is not stainable with the Gram technique [15]. *Coxiella burnetii* is a microorganism with a very high infection capacity; a single germ is capable of causing infection [27]. This bacterium presents a phenomenon called “antigenic phase variation”. It is a molecular phenomenon that is produced by modification in the complexity of the membrane LPS, which will cause a difference in virulence [28]. The main reservoirs of *C. burnetii* are cattle, sheep, and goats, but infections were detected in other animals such as domestic mammals, marine mammals, reptiles, birds, and ticks. *Coxiella burnetii* is most abundant in birth products and in the urine, feces and milk of infected animals. Transmission to humans most commonly occurs through inhalation of aerosolized bacteria from the placenta (delivery or abortion), feces, or urine of infected animals. Human-to-human transmission is extremely rare [15,28,29]. Other routes of transmission of the disease are oral transmission after the ingestion of contaminated raw dairy products, transfusion of blood products, sexual transmission, and professional exposure, as in the case of pathologists or microbiologists [30,31]. In humans, the average incubation time is 18 days (between 7 and 32 days) [29]. Infected animals are usually asymptomatic. Spontaneous abortions, endometritis, mastitis, and infertility are the only signs that can be observed [15]. In humans, Q fever can manifest as an acute disease usually as a self-limited febrile illness, pneumonia, or hepatitis. It may also occur as a persistent focalized infection with endocarditis [32]. In humans, the diagnosis of Q fever is mainly made by serology, microbiological cultures, or PCR tests [28]. The European Union (EU) One Health Zoonoses Report indicates that 950 human cases of Q fever have been reported in the EU in 2019 [5]. According to CDC, a total of 212 cases of Q fever have been reported in the US in 2019, including 178 human cases of acute illness and 34 cases of chronic Q fever disease [6]. In Africa, seroprevalence rates in humans varied from 1% in Chad to 16% in Egypt [15]. Between 2007 and 2010, there was an outbreak of Q fever in the Netherlands, with more than 4000 reported human cases and an estimation of probably more than 40,000 total human cases [15]. The highest prevalence recorded is in Cayenne, French Guiana, where *C. burnetii* causes 24% of community-acquired pneumonia [33].

## 3. Brucellosis

Brucellosis is caused by the intracellular pathogens from genus *Brucella* [34]. *Brucella* spp. can multiply within phagocytic cells with human beings as end hosts [35]. Four species of *Brucella* can infect humans: *B. abortus, B. canis, B. melitensis*, and *B. suis*. Of these species, *B. melitensis* is the most commonly isolated from ruminants [16]. In sheep and goats, which are the main hosts of *B. melitensis*, the bacterium causes impaired fertility and abortions [36]. Brucellosis in cattle is caused by *B. abortus*, which can be easily transmitted to humans. Brucellosis may be transmitted to humans through contaminated food and dairy products, occupational contact, or inhalation of infected aerosols [16]. Another important route of infection is the contamination of mucous membranes or open wounds with fetal fluids, making veterinarians, farmers, and abattoir workers the most vulnerable to infection. In other cases, transmission from animals to humans is mainly associated with drinking contaminated milk [37]. Human-to-human transmission takes place through lactation, sexual intercourse, and tissues such as blood transfusion and bone marrow transplantation [38]. Brucellosis in humans has several, often non-specific, presentations, including a systemic syndrome (fever, sweat, chills, and fatigue), but also some located presentations (epididymoorchitis and spondylodiscitis). Severe forms of this disease are neurobrucellosis and endocarditis [38]. Brucellosis is one of the most frequent zoonosis in many parts of the world. However, this disease mainly affects humans in developing countries, as it is effectively controlled in developed countries [16]. Brucellosis is an endemic zoonosis for the Middle East, the Mediterranean rim, Asia, Africa, and South and Central America. These are regions with a very high consumption of dairy products and insufficient animal health care [37]. Around 500,000 cases of brucellosis in humans are reported worldwide each year [36]. However, true incidence is estimated to be 5,000,000 to 12,500,000 cases annually [39]. Seroprevalence by country in sub-Saharan Africa is 24.1% and 31.82% in Nigeria, 17% in Uganda, 7.7% in Tanzania, 3.8% in Chad, and 3.3% in the Central African Republic [40]. Brucellosis is a major economic problem in African countries such as Nigeria. In this country, high losses are generated for cattle producers due to stillbirths, reduced calving percentages, medical costs, births of weak calves, culls due to infertility, and the loss of man-hours in infected people. Furthermore, wastes in meat and dairy production are estimated at USD 224 million per year. To compare, in the Republic of South Africa, the losses due to brucellosis are USD 37.5 million and in the USA, they equal to USD 800 million per year [41]. According to the EU One Health Zoonoses Report, 310 human cases of brucellosis have been reported in the EU in 2019 [5]. As reported by the CDC, areas currently listed as high risk of brucellosis are the Mediterranean Basin (Portugal, Spain, Southern France, Italy, Greece, Turkey, and North Africa), Mexico, South and Central America, Eastern Europe, Asia, Africa, the Caribbean, and the Middle East [6]. In the United States, brucellosis is a rare disease, with 80–120 cases reported annually [6]. Syria has the highest number of human brucellosis with 1603.4 cases per 1,000,000 individuals. This is followed by Mongolia (3910), Iraq (268.8), Tajikistan (211.9), Saudi Arabia (149.5), and Iran (141.6) [39]. In China, brucellosis is also an important public health threat. In 2014, 4.2 cases/100,000 people were reported [42].

## 4. Tuberculosis Caused by *Mycobacterium bovis* and *Mycobacterium caprae*

**Figure 2:**
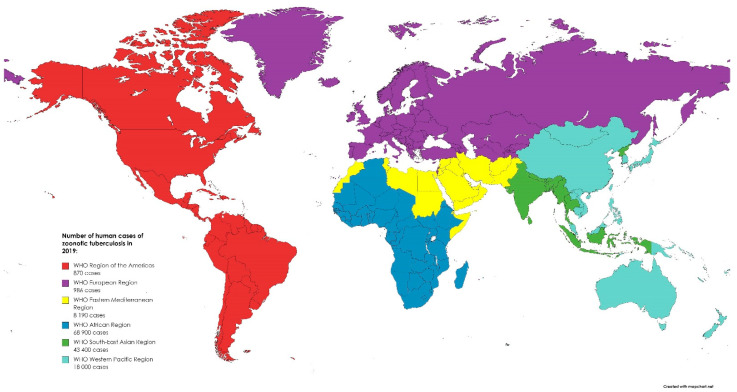
The global distribution of zoonotic tuberculosis human cases in 2019. Created based on data from [47].

*Mycobacterium caprae* and *Mycobacterium bovis* are members of the *Mycobacterium tuberculosis* complex and cause tuberculosis (TB) in animals and humans. *M. caprae* is isolated not only from goats but also from sheep, red deer, cattle, wild boar, the Siberian tiger, camel, bison, and humans [17]. *M. caprae* causes lesions and diseases like that of *M. bovis* but occurs only in a low proportion of human TB cases. Moreover, *M. caprae* is evolutionarily older than its epidemiological twin, *M. bovis*. This bacterium is not globally distributed but primarily restricted to European countries [43]. On the other hand, the most common host of *M. bovis* is cattle, but other mammals, such as marsupials, carnivores, pinnipeds, lagomorphs, rodents, and some avian species, could be also infected [44]. The main route of TB transmission in animals is via aerosol by the droplet nuclei generated during coughing and sneezing. Humans may be also infected through milk, dairy products, and by eating meat from infected animals [45]. The disease is manifested in humans by fever, fatigue, arthralgia, and muscle pain, and variety of other symptoms depending on the part of the body affected by the disease. WHO reported that in 2016, there were 147,000 new cases and 12,500 people died due to TB, but with no information of the potential zoonotic origin [18]. *M. bovis* is responsible only for 3.1% cases, with the exception for Tanzania, in which it reached 16% of TB in humans possibly due to poor zoohygienic conditions [46]. In 2019, most of the zoonotic TB human cases occurred in Africa (50%) and South-East Asia (31%). Globally, there were 140,000 human cases of zoonotic TB. However, the uncertainty level is estimated to be 69,800 to 235,000 [47]. While in Europe, TB is a rare infection with 147 confirmed cases in humans reported in 2019 in the EU. Between 2015 and 2019, 918 cases of TB were confirmed in the EU, including 54 caused by *M. caprae* [5]. The global distribution of zoonotic TB human cases in 2019 is presented in Figure 2. The most effective way to eliminate TB in farm animals is through implementation of eradication programs. In developed countries, infection with *M. bovis* is not common in cattle. This is related to compulsory tuberculin testing, the pasteurization of milk, and the removal of positive reactors [48]. Before the routine application of milk pasteurization in the United Kingdom (UK), *M. bovis* was isolated from 8% of churn milk samples from 3000-gallon tankers in 1945 [49]. In the 21st century, only 315 cases of human TB have been reported in the UK over a 10-year period [50]. However, it is important to note that cattle can become infected from wild mammals. This can also have an impact on the eradication of the disease. To date, the following have been identified as reservoirs of the mycobacteria around the world: brushtail possum and badger, European bison, African buffalo, wild boar, and white-tailed deer, among many others [51]. Research shows that Michigan deer may have infected surrounding cattle. Data indicate that while recording cases of the disease in wild deer between 1975 and 1994, infected animals were found in sixteen domestic cattle herds in four counties in the north-western part of the state [52]. Transmission from humans to cattle is also possible. *M. bovis* is usually transmitted directly by inhalation but also indirectly by hay and bedding contaminated with urine. In the Netherlands, humans were the source of transmission for 50 cattle herds [53]. Zoonotic *M. bovis* infections are mainly a problem in undeveloped countries. In the developing countries, due to the lack of control of zoonotic products, poor production hygiene, and outbreaks of other diseases (e.g., AIDS), the pathogen will continue to persist and remain a real challenge for public health in the future.

## 5. Shiga Toxin-Producing *Escherichia coli* (STEC)

Shiga toxin-producing *Escherichia coli* (STEC) are emerging foodborne pathogens whose infection in humans is associated with varying clinical manifestations, including diarrhea, hemorrhagic colitis, and (occasionally fatal) hemolytic uremic syndrome (HUS) [54]. Cattle is recognized as the major STEC reservoir [55], although sheep and goats [56] may be also important sources of this pathogen. The dominant transmission route includes ingestion of contaminated food or water, direct contact, or exposure to a communal environment. Most STEC-colonized animals are asymptomatic but some STEC strains may be associated with diarrhea in neonatal calves [57]. The prevalence of STEC strains in cattle varied from 0.4 to 74.0% according to the data collected from Canada, the US, Brazil, Spain, Italy, Germany, Denmark, Japan, and the UK in the year 2005 [58]. For example, according to Ballem et al. [59], the prevalence of STEC in Portugal was 45% in heifers and 16% in lactating cows. In 2019, 29 EU/EEA countries reported 8313 confirmed cases of STEC infection [5]. In recent analyses, beef and fresh produce (fruit and vegetables) were found to be the most important sources of STEC infections in Europe, each responsible for 30% of human cases [5]. To compare, in 2017, a US state and regional public health laboratories confirmed 6034 STEC infections with the O157 as the dominant serogroup [6]. Similarly, the most common transmission mode was foodborne (43% of cases), including consuming vegetable row crops, beef, dairy, and fruit [60]. Interestingly, there is a study reporting that Shiga toxin-encoding genes were detected in 21 (3.4%) of 621 farmers and 15 (7.6%) of 198 slaughterhouse workers’ stool samples, which suggests that dairy farmers and beef slaughterhouse work places are group of special risk [61]. On the other hand, the study performed by Bai [62] evaluated the prevalence of STEC from retail raw meats collected from two geographical regions in China and the results revealed that 166 out of 853 samples were Shiga toxin-positive; 63 STEC isolates were recovered from 58 Shiga toxin-positive samples, including pork (4.4%, 14/318), beef (11.0%, 21/191), mutton (20.6%, 26/126), chicken (0.5%, 1/205), and duck (7.7%, 1/13). Good hygiene practice during food and water processing may decrease the risk of STEC transmission. Undercooked meat including beef and raw milk should be excluded from the diet.

## 6. Trichinellosis

Trichinellosis is a foodborne, zoonotic disease caused by nematodes of the genus *Trichinella* [19]. This genus consists of 12 taxa, including three genotypes and nine species [63]. *Trichinella* spp. can infect many of the animal species, mainly carnivores and omnivores such as pigs, wild boars, cats, wolves, rodents, and humans [19]. Infection in humans is induced by ingestion of raw or undercooked muscle tissue containing encysted larvae and can be divided into two phases: intestinal and muscular [64,65]. Depending on the stage of infection, the symptoms diarrhea and abdominal pain (intestinal phase) may be observed at first, and fever, myalgia, myocarditis, facial oedemas, and encephalitis may be observed later [66]. The main source of infection for humans is pork and game [67]. An atypical case of trichinellosis was reported in 2014. A 51-year-old woman who was on a vegetarian diet reported to the hospital with a bilateral swelling of the legs, myalgia, and muscle weakness. Histological examination of muscle biopsy showed that the *Trichinella* sp. worm was the cause of these complaints. The patient admitted that she had handled meat from a wild boar 1 month before the first hospitalization. Examination of the meat confirmed the presence of *T. britovi* [68]. *Trichinella* spp. is spread worldwide [69]. In 2019, 96 confirmed cases of trichinellosis in humans were reported in 12 European countries (Austria, Bulgaria, Croatia, France, Germany, Italy, Latvia, the Netherlands, Poland, Portugal, Romania, and Spain). That induced a growth of the EU notification rate to 0.02 per 100,000 population compared with 2018 (0.01 per 100,000 population). In fattening and breeding pigs kept under controlled housing conditions, no infection with *Trichinella* spp. was reported, but in swine that were not keep under controlled housing conditions, 218 fattening pigs (out of 139.6 million) and 1 (out of 5.6 million) were *Trichinella*-positive. Infected swine came from free-range and backyard pigs reared in rural regions of Europe. In total, 1.368 (0.08%) hunted wild boars were tested positive for the presence of this parasite [5]. However, these data can be undervalued. Vieira-Pinto et al. [70] pointed out that 86 out of a total of 100 inquired hunters admitted they use hunted meat for private purposes. In total, 93% of those declared that they also have sold part of the meat, the majority (80%) without prior testing for *Trichinella* spp. [70]. This creates a great risk of infected meat circulation. Reports from the US show that during 2008–2012, 90 cases of trichinellosis in humans were notified from 24 states and the District of Columbia [6]. There were no positive results in 85 million samples taken from pigs in the controlled system, while in the non-controlled system, there were 10–20 confirmed pig cases (15 million samples taken) per year [64]. In order to prevent infection, raw or undercooked pork and wild game should not be eaten. In the EU, all meat that is going to be placed at the market should be examined for presence of *Trichinella* larvae [71].

## 7. Yersiniosis

Yersiniosis is a foodborne zoonosis caused by bacteria which belong to the *Enterobacteriaceae* family, namely of the *Yersinia* genus [5,72]. *Yersinia enterocolitica* is the main etiological factor of this disease [20]. Infections associated with *Y. pseudotuberculosis* are less common [20]. *Y. enterocolitica* was divided into six biotypes (1A, 1B, 2, 3, 4, 5, and 6) and over 70 serotypes. All biotypes, except the first one, emerged to be pathogenic, especially 1B [73]. Bioserotype 4/O:3 is the most common bioserotype associated with infections in humans [73] and pigs are its major reservoir [74,75]. Swine mostly developed no signs of infections, which is what makes them asymptomatic carriers [75]. People can get this disease by eating raw or undercooked pork [76,77] but infections due to ingestion of dairy products, seafood, vegetables, or drinking water are also possible [60,78,79,80]. In humans, infection usually affects children, immuno-compromised patients, and elderly. Clinical symptoms include fever, vomiting, abdominal pain, and bloody diarrhea [72,80]. According to the EU One Health Zoonoses Report, yersiniosis was the fourth most frequently reported zoonosis in 2019, with a stable trend in 2015–2019 (6961 confirmed cases) on the territory of member states. During 2019, seven countries (Denmark, Finland, France, Germany, Poland, Lithuania, and Sweden) reported 15 foodborne outbreaks, yielding 149 illnesses [5]. The CDC reports that *Y. enterocolitica* may be responsible for nearly 117,000 illnesses and 35 deaths in the United States each year [6]. Duan et al. [81] assessed the prevalence of yersiniosis in children with diarrhea in China from 2010 to 2015. In children < 5 years old, the prevalence was 0.59% (43 out of a total of 7304 patients with diarrhea). In Beijing, the presence of *Y. enterocolitica* was confirmed in both children (13/2127) and adults (2/1904) that had reported to the hospital with diarrhea [81]. To avoid infection, raw or undercooked pork should not be eaten, especially by children.

## 8. Salmonellosis

*Salmonella* is a large, ubiquitous genus of Gram-negative, rod-shaped, facultative anaerobic bacteria belonging to the family of *Enterobacteriaceae* and is responsible for zoonotic infections of global significance. It can be persistent in dry environments as well as in water for months [82]. There are two main species distinguished: *S. enterica* (which includes more than 2600 known serovars) and *S. bongori*. The majority of variants of S. enterica are motile by the means of flagella but the most important virulence factors are invasion and intracellular replication [82]. *Salmonella* was first isolated in 1884 by an American bacteriologist, D. E. Salmon, from porcine intestine [24] and in the 1980s, the first pandemic of *S. enterica* s. *Enteritidis* emerged due to contaminated poultry products [83]. *Salmonella* sp. may cause clinical disease in livestock or subclinical infections in asymptomatic animals (carriers), such as dogs and cats, which transmit and contaminate the environment of food-producing animals. A very important role is played by vertical transmission, especially in the poultry and bovine reproduction sector, but pests are also a significant vector of the germ [25,82]. An infection in humans can occur after drinking contaminated water or ingesting uncooked contaminated eggs, milk, and meat originating from poultry, cattle, or swine, although there have been reports about other foods, including vegetables contaminated by manure and ready-to-eat foods that caused infection. Human-to-human transmission through the fecal–oral route and infection after direct contact with infected animals, their feces, and the environment are less common, although still significant. What is a concern is that *Salmonella* can pass through the entire food chain, starting from animal feed contaminated by manure and primary production to the table in households, food services, and institutions (farm-to-fork continuum) [6,84]. EFSA reported that salmonellosis after campylobacteriosis was the second most often reported gastrointestinal infection in humans. In 2019, 87,923 cases were confirmed in the EU [5]. According to OIE, salmonellosis qualifies as one of the most common foodborne bacterial diseases in the world. Human infections caused by *Salmonella* species are most frequently caused by *S. Enteritidis* and *S. Typhimurium*, which are normally found in the intestines of humans and animals, as they are the main reservoir of these bacteria [3,24]. A distinction is made between three major diseases caused by *Salmonella* in humans, namely non-invasive non-typhoidal salmonellosis, invasive non-typhoidal salmonellosis, and typhoid fever, but in general, salmonellosis manifests with acute enterocolitis accompanied by inflammatory diarrhea, abdominal pain, fever, nausea, and vomiting in humans [85]. Most cases of the disease are underdiagnosed, turning salmonellosis into a disease that contributes to the deaths of thousands of people worldwide, especially in economically underdeveloped countries [82]. The majority of foodborne outbreaks was caused by *S. Enteritidis*. The highest number of reported domestic salmonellosis cases was in the Czech Republic, followed by Hungary, Latvia, Lithuania, Malta, Portugal, Poland, Slovakia, and Spain, while the highest proportions of infections related to travelling was in Nordic countries. The most frequent travel-associated *Salmonella* came from Turkey, Egypt, Thailand, India, Spain, and Greece [5]. According to data published by the CDC, *Salmonella* causes about 1.35 million illnesses and 420 deaths every year in the US and most of them are caused by contaminated food [6]. In 2017, Stanaway et al. estimated that 535,000 cases of non-typhoidal salmonellosis occurred with the highest rates in sub-Saharan Africa [86]. *Salmonella* serotypes and prevalence can vary significantly depending on geographic factors, thus surveillance and identification of mentioned bacteria found in both humans and animals (especially poultry, the major zoonotic source of the disease in people) must be conducted to develop a control program for a given area [3]. *Salmonella* prevention and control in poultry production employs principles of good agricultural practice, hazard analysis, and critical control point (HACCP) principles, as well as other measures (such as vaccination, culling, and further processing of animal products), none of which alone will provide effective control of the described pathogen. Antimicrobials should not be used as the treatment effectiveness is limited. Furthermore, antibiotic resistance is on the rise and potential disruption of the normal intestinal flora of birds may be created, increasing the likelihood of *Salmonella* colonization [3,87], while consumers should avoid eating raw eggs or undercooked poultry meat.

## 9. Campylobacteriosis

*Campylobacter* spp. are Gram-negative, microaerophilic, and thermophilic bacteria of a spirally curved shape and primarily motile by means of a polar flagellum [88]. They exhibit chemotaxis, adhere to and invade host cells, produce toxins, and form a biofilm allowing bacteria to survive in a hostile environment [89,90]. The first microorganism of the *Campylobacter* genus was described in 1886 by Theodor Escherich, a pediatrician who isolated it from the stools of children suffering from diarrhea [89]. *Campylobacter* spp. can adapt to environmental stresses. They develop tolerance to acidic environment, UV light, desiccation, and salt. They can also show thermotolerance and osmotolerance, and form biofilm [88]. *Campylobacter* strains are widely distributed in nature and gastrointestinal tracts of the majority of warm-blooded animals, with birds, cattle, and pigs being the main reservoir of the pathogenic germs of zoonotic potential [89]. These ubiquitous bacteria are transmitted from animals to humans directly or via the food chain by raw and undercooked poultry meat, as broilers may be asymptomatic carriers of pathogenic *Campylobacter* strains [88,91]. In poultry, pathogens spread through an oral–fecal route or by vertical transmission. It is not frequently cross-contaminated from environment to the animal [89] but insects, amoebae, yeasts, and molds have been identified as vectors of horizontal transmission [92]. Moreover, contaminated water and animal products such as milk, dairy products, and red meat may pose as potential sources of infection for humans [93]. People can also become infected by seafood, fruits, and vegetables contaminated by pathogens through contact with animal feces or soil or through ready-to-eat foods, a lack of hygiene in food preparation, or by contact with animals and their feces [6]. *Campylobacter* infection (campylobacteriosis) is a bacterial infection which most commonly causes gastroenteritis. *C. jejuni* and *C. coli* are the major causes of foodborne infection [94] and are often found in poultry [89]. In most cases, the clinical course of infection in humans is self-limiting, although some individuals may develop autoimmune disorders, cardiovascular disease, and sepsis [93]. Infected people may experience additional complications, such as IBD (Inflammatory Bowel Disease), reactive arthritis, or neuropathies (i.e., Guillain-Barré syndrome). A life-threatening infection can affect those with a weakened immune system [6,91]. Most humans affected by campylobacteriosis showed symptoms such as watery or bloody diarrhea, abdominal pain, fever, headache, and vomiting [93]. Campylobacteriosis affected 220,682 people in 2019 and has been the most reported zoonotic gastrointestinal disease in the EU since 2005. According to the EFSA, most of the human campylobacteriosis domestic cases were described in the Czech Republic, Hungary, Latvia, Malta, Poland, Portugal, Romania, and Slovakia. Moreover, numerous travel-associated cases were reported by Scandinavian countries, having brought infection from Spain, Greece, and Italy. Outside of UE borders, many cases were recorded in Turkey, Thailand, and Morocco [5]. Many *Campylobacter* infections are undiagnosed or unreported, thus the total number of them is underestimated [6,91]. The prevalence of campylobacteriosis in humans remained relatively stable from 2015 to 2019 [5]. The disease is common in underdeveloped countries [6]. The widespread occurrence of *Campylobacter* spp. in poultry production and processing could be contained by improving biosecurity systems and applying effective intervention strategies. The importance of the measures undertaken is great as there is no effective critical control point in processing raw poultry meat [92]. Nonetheless, awareness should be raised on increasing antimicrobial resistance as well as on the prevalence of pathogenic bacteria in the gastrointestinal tract induced by antibiotic growth promoters (AGP) administered to poultry [91]. Alternatives include the use of probiotics, plant-based antimicrobials, metal oxide nanoparticles, bacterial synergism, or active packaging to maintain the best possible product freshness and quality [94].

## 10. Influenza

Avian influenza viruses (AIV) belong to the Orthomyxoviridae family and are divided based on molecular differences into types (A, B, C, or D). Birds are the natural reservoir of influenza A virus, which may cross the species barrier and cause zoonotic infections in humans [2,6]. AIVs are classified into two pathotypes based on their virulence in chicken. Low pathogenic avian influenza (LPAI) strains primarily affect ducks and chickens [83], and cause mild disease with respiratory symptoms (i.e., coughing, nasal and ocular discharge, and swollen sinuses), decreased egg production, and infertility of different backgrounds, but morbidity and mortality are rather low. Infections in birds are most commonly caused by H9N2 in the poultry market of Asia, North Africa, and the Middle East [84]. Strains of highly pathogenic avian influenza (HPAI) virus primarily affect chickens and turkeys [83], in which they cause severe, systemic disease with high morbidity and mortality. Since 1959, numerous worldwide outbreaks of the disease in poultry and wild birds have been caused by HPAI H5 and H7 viruses carrying diverse NA subtypes [95]. Transmission of AIVs between birds is followed by direct contact (ingestion or inhalation) with saliva, respiratory secretions, and feces of the infected individual. Indirectly, the virus spreads through contact with contaminated surfaces, such as equipment or clothes, as AIVs have the ability to survive for a long time in low temperatures [3,6]. Transmission between farms occurs in cases of violation of the biosecurity rules [96]. Additionally, swine play an important role in the disease epidemiology since influenza A viruses show the high ability to reassort [6,97]. Human infections may occur after direct contact with infected birds, ingestion of raw or undercooked poultry products, and after human-to-human transmission [96]. The spread of the disease is encouraged by growing globalization, international trading (among others, live bird markets), and migration of wild birds [3]. Infection in humans may manifest as a mild upper respiratory infection causing fever, headache, and cough, along with conjunctivitis and gastrointestinal problems [6]. However, progression to severe pneumonia, acute respiratory distress, multi-organ failure, and shock may be rapid. Fatal cases of the disease have also been reported [84]. Most severe illnesses with the highest mortality among humans are caused by viruses originating from HPAI A(H5N1) and LPAI A(H7N9) infections [83,84]. First human infections with the HPAI A(H5N1) virus were reported in Hong Kong in 1997 during an outbreak in poultry [83]. Since then, the virus spread across Asia to Europe and Africa, causing numerous infections and deaths in poultry and humans, pitting the economy and international trade [84]. LPAI A(H7N9) virus infections were first reported in 2013 in China, causing 1500 human infections and many deaths, and affecting the population of poultry [83,84]. Moreover, in 2016, LPAI A(H7N9) evolved to HPAI, thus causing more advanced clinical signs and more severe disease consequences [83]. Furthermore, human infections with other types of avian H5-H10 influenza viruses have also been recorded, including the A(H5N8), A(H7N7), and A(H9N2) viruses [83,84]. Avian influenza cases of human infections in the Western Pacific Region are reported weekly by WHO. There were 239 (of these, 134 fatal) reported cases of the disease caused by A(H5N1) virus since 2003. The last reported case was in 2020. There also were 1568 (including 616 fatal) confirmed human infections with A(H7N9) virus reported by WHO from early 2013 to 2021 [84]. Control and prevention measures of HPAI H5 or H7 avian influenza include culling of infected flocks and quarantine of exposed flocks. Nearby or linked-to-the-infected-flock birds should be under observation [6]. The use of vaccines and adequate management strategies is possible to control HPAI viruses’ outbreaks [96]. However, the most important action to be taken for reducing the risk of human infection is to control the circulation of avian influenza viruses in poultry, especially since some of them (such as A(H5) and A(H7N9) viruses) may persist in poultry populations and their control requires good coordination between animal and public health authorities [84].

Swine influenza is caused by influenza A virus (IAV), called swine influenza virus (SIV). IAVs are categorized into 18H and 11N subtypes based on features of two proteins: hemagglutinin (HA) and neuraminidase (NA) [98]. IAVs can infect not only pigs but also people; one of the IAVs, namely (H1N1)pdm09 new triple-reassortant virus, caused a pandemic in the human population in 2009 [99]. (H1N1)pdm09 is the product of reassortments among multiple swine influenza virus lineages: its NA and M genes were derived from the Eurasian avian-like swine H1N1 influenza virus (EAsw SIV), while its other genes were from the triple-reassortant (TRsw) SIV with PB2 and PA derived from avian H1N1, PB1 from human H3N2, and HA, NP, NS, NA, and M from classical swine H1N1 [21]. Swine influenza virus is a single-strand negative-sense RNA virus which belongs to the Orthomyxoviridae family. Pigs can become naturally infected with swine but also avian and human influenza viruses due to the expression of both sialic acid (SA) receptor types in the respiratory track. This creates a risk of new reassortants of influenza virus and makes swine a ‘mixing vessels’ [22]. Swine farmers, veterinarians, and pork processing workers are in the group of increased risk of SIV infection or infections caused by reassortants created in the swine respiratory tract [23]. Infection in humans is induced by contact with respiratory discharges or inhalation or exhalation of sick pig aerosol [100]. However, there is a study which suggests that people can become infected with SIV without close, direct contact with pigs. This implies secondary transmission of SIV by person or fomite [101]. Clinical signs of influenza are similar in human and pigs, inducing symptoms from the respiratory system (sneezing, coughing, and difficult breathing) as well as fever, lethargy, and decreased appetite [102,103]. Sometimes infection can be fatal, mostly in children or in individuals with decreased immunity. SIV circulates in pig populations worldwide [98]. Currently, three main subtypes of IAV, namely H1N1, H3N2, and H1N2, are distributed in the global swine population [104]. Vaccination is the main strategy to prevent infection, in both human and pigs. However, due to a variety of subtypes of SIV in the worldwide population, efficient control of the disease may be challenging [105]. Research conducted by Saunders-Hastings et al. [106] demonstrated that frequent hand- washing ensures a significant protection level against infection with the 2009 pandemic influenza, in contrast to facemasks which provided a non-significant protective effect [106]. However, a study performed by Wong et al. [107] showed that the combination of these two measures is an effective strategy to prevent disease [107]. Ayim-Akonor et al. suggested that poor biosafety management may enable easier cross-species transmission of influenza virus between humans and pigs [108].

## 11. Listeriosis

Listeriosis is an important emerging zoonotic disease caused by the intracellular, psychrophilic, Gram-positive bacterium *Listeria monocytogenes* (*L. monocytogenes*). It is able to survive in the environment for a long time not only because it withstands large-scale temperatures (−1.5 to 50 °C) and adapts to adverse environmental conditions (high concentrations of salt, oxygen-limiting conditions, or low pH), but also due to its capability of causing asymptomatic infections in animals (including birds) [109,110]. Like *Campylobacter* sp., *L. monocytogenes* can form biofilms on a variety of surfaces, resists desiccation, and exhibits osmoadaptation. Moreover, it shows resistance to sanitizing agents [110]. It affects human health by being transmitted orally via contaminated food [109]. Humans may become infected after ingesting uncooked or ready-to-eat foods (i.e., meat, milk, dairy products, and vegetables) [111], and after drinking contaminated water [112]. Most common manifested symptoms of localized infection are diarrhea, abdominal pain, and flu-like symptoms. In contrast, systemic disease may be manifested by fever, headache, encephalitis, meningitis, and liver abscesses. Listeriosis is particularly dangerous for pregnant women as it may cause abortion, premature births, or stillbirths. Neonatal infections, pneumonia, and even sepsis may occur in the neonates [110]. The EFSA reports that in the 2019, 2621 confirmed human listeriosis cases occurred in the EU. It is worth noticing that the listeriosis proportion of hospitalized cases was the highest of all zoonoses under EU surveillance. Nine strong-evidence foodborne outbreaks were identified in the EU. Most of them was caused by meat and meat product consumption [5]. In 2018, WHO estimated that the incidence of listeriosis is 0.1 to 10 cases per 1 million people per year worldwide, which makes it a relatively rare disease. However, the infection is followed by a high rate of deaths; *L. monocytogenes* infection is therefore an important public health concern [84]. Since *L. monocytogenes* is ubiquitous, controlling its presence in the food production environment is crucial [112]. Good Hygienic Practices (GHP), Good Manufacturing Practices (GMP), as well as the principles of Hazard Analysis Critical Control Points (HACCP) of the food safety management system should be implemented along the entire food chain. It is essential to respect the shelf-life and storage temperature of ready-to-eat foods, as well as to pasteurize or cook food before eating [84]. Since the 1990s, the prevalence of *L. monocytogenes* in many food categories decreased due to improved food-chain control measures [112].

## 12. Glanders

Glanders is an infectious and zoonotic disease caused mainly by *Burkholderia mallei*. This Gram-negative and host-restricted bacterium belongs to the *Burkholderia pseudomallei* complex together with *B. pseudomallei*, which causes melioidosis [113]. Solipeds such as horses, donkeys, and mules, as well as humans, are susceptible to infection. Ungulates are the natural reservoir of the disease and the source of infection. Glanders is a rare disease of humans but infection can occur through direct or undirect contact with an infected animal and their secretions. The most vulnerable professional groups are veterinarians, horse caretakers, laboratorians, equine butchers, and abattoir workers. Human-to-human transmission is also rare. *Burkholderia mallei* can invade the host through the mucous membranes, gastrointestinal tract, and the integument. The symptoms of glanders are similar in humans and animals. This disease is characterized by ulcerating nodular lesions of the skin and the mucous membrane, together with the presence of generalized symptoms such as fever, malaise, depression, cough, anorexia, and weight loss. The main problem in the serological diagnosis of this disease is the occurrence of false-negative and false-positive results, which causes problems for international trade in Equidae [114,115,116]. *Burkholderia mallei* has been used as a biological weapon in battlefields for centuries. This bacterium belongs to the Tier 1 biological agent with the USA Federal Select Agents Program due to its high infectivity, degree of incapacitation, and resistance to treatment [115]. Glanders has been eradicated in many countries but is still present in Africa, Asia, the Middle East, Central America, and South America. Due to globalization, the disease is recognized as re-emerging [117]. The disease has been eradicated in North America, Australia, and Europe through testing and eradication of infected animals, along with strict import control rules for Equidae. Glanders is an OIE-listed disease as described in the Terrestrial Animal Health Code of the World Organisation for Animal Health (OIE) and any disease outbreak must be notified to the OIE [6]. From 1992, equine glanders cases were reported in countries such as Pakistan, Brazil, India, Iraq, Iran, Turkey, Bahrain, Kuwait, Russia, and China [114]. Germany notified the occurrence of the disease in horse in a limited area in 2014/2015 [3].

## 13. West Nile Fever

West Nile Virus (WNV) is a positive-stranded RNA virus belonging to the family Flaviviridae and genus Flavivirus. The virion consists of an envelope surrounding an icosahedral capsid [118]. The pathogen causes neurological disease mainly in humans and Equidae, called West Nile fever. Diseases are also reported in a wide range of wild and domestic animals, including birds, reptiles, and mammals [119,120]. WNV was detected in snakes and antibodies against WNV were found in farmed crocodiles and alligators [121]. This virus is globally distributed and maintained by a complex transmission cycle involving multiple species of mosquitoes and birds [119,120]. Birds are reservoir hosts and migratory birds play an important role in the virus transmission between continents [122]. The virus in the blood of birds with viremia may be transmitted by mosquitoes to humans, horses, and other animals [123]. WNV causes a wide range of symptoms in humans, from asymptomatic or mild infection to severe and often fatal central nervous system infection [124]. The first ever recorded case of WNV infection in humans occurred in Uganda in 1937 [123]. Since 1999, when the first case of human WNV infection was confirmed in the Americas, there have been more than 48,000 cases, 24,000 neuroinvasive cases, and more than 2300 deaths through 2019. During this time, more than 28,000 cases of the disease have been reported in horses. High mortality was recorded in more than 300 bird species, which was the cause of a large population decline in 23 of them. More than 5000 cases in human have been reported in Canada [119]. For 2019, 443 WNV infections in humans were reported in the EU. In 2018, these cases were as many as 1615 [5]. The highest number of infections in humans is recorded in Greece, Germany, and Italy. In contrast, a total of 153 animals’ outbreaks have been reported in the EU in 2019, including 53 in birds and 100 in horses [5]. To avoid West Nile fever, people should predominantly prevent mosquito bites using different methods including proper clothing, using effective repellent, and avoiding areas with confirmed WNV presence.

## 14. Melioidosis

Melioidosis is a tropical and zoonotic disease of animals and humans caused by the Gram-negative, motile, environmental, and opportunistic bacterium *B. pseudomallei*. The first case of human melioidosis was described in Australia in 1950. Melioidosis occurs in various animal species, including horses, mules, cats, rats, rabbits, dogs, deer, camelids, cows, parrots, koalas, kangaroos, and human and non-human primates, but it is most commonly found in sheep, goats, and pigs. The estimated incidence rate of melioidosis among goats in Thailand (endemic region) from 2006 to 2010 was 1.63 per 100,000 population per year [125]. Cases of the disease in animals in non-endemic areas are sporadic, as in humans [126]. The clinical forms of the disease in animals vary according to the species of animal but the most frequent forms are acute fulminate septicaemia, local infection, subacute illness, chronic infection, and subclinical disease [126]. The predominant transmission route of the disease is percutaneous inoculation after exposure to wet-season soils or water. Less common routes of infection are inhalation and ingestion of *B. pseudomallei*-contaminated matter (e.g., water) and vertical, zoonotic transmission, or transmission to offspring through milk from mothers with mastitis and sexual intercourse. The most common clinical forms of melioidosis are acute pneumonia and the cutaneous form with a solitary lesion at the site of inoculation. Visceral abscesses are also frequently found in the spleen, liver, adrenals, and kidneys. Nerve form, osteomyelitis, septic arthritis, mycotic aneurysms, pericarditis, mediastinal masses, and scrotal abscesses are noted very rarely. The disease develops most commonly in people with comorbidities, such as diabetes mellitus, alcohol abuse, and immunosuppression. Due to the multitude of clinical forms, melioidosis is defined as “the Great Mimicker” [127,128,129,130]. Recent studies indicate that the disease is widespread. Around 165,000 cases of the disease are recorded annually, with human mortality rates as high as 89,000 deaths worldwide [128]. Melioidosis is endemic in North Australia, Southwest Asia, India, and China. Other regions where the disease occurs are other areas of Asia, Central and South America, Africa, and the Pacific and Indian Oceans [127,129]. An increase in the incidence of melioidosis is observed during the rainy season, when both humans and animals are more exposed to wet soil [126]. In Europe and North America, the disease is mainly spread by animal transport from endemic areas and by tourists. Between 2000 and 2018, 77 cases of imported melioidosis have been reported in Europe [129]. According to the CDC, in the period from March to July 2021, *B. pseudomallei* was detected in aromatherapy products and four cases of melioidosis in humans have been reported [6]. 

## 15. Conclusions

Human and animals’ health are intimately connected since they share a communal environment. Consequently, pathogen transmission is possible and it may occur via direct and/or indirect contact, including consuming products of animal origin. In the case of confirmed infection derived from animals or animal products, a comprehensive approach should be applied. Thus, in the suspicion of any zoonoses, the medical and veterinary doctors should closely cooperate to protect public health and work in accordance with the One Health conception.

## Figures and Tables

**Table 1:** A brief summary of the most significant livestock-associated zoonoses.

Disease	Aetiological Agent	Human Symptoms	Transmission Route	Epidemiology	References
Q fever	*Coxiella* *burnetti*	Self-limited febrile illness, pneumonia, hepatitis, and endocarditis	Inhalation of aerosolized bacteria, ingestion, transfusion of blood, and sexual transmission	EU—950 human cases in 2019 USA—178 human cases in 2019	[5] [6] [15]
Brucellosis	*Brucella* *abortus,* *B. melitensis,* *B. canis,* *B. suis*	Systematic syndrome (fever, sweat, chills, and fatigue), located presentations (epididymoorchitis and spondylodiscitis), neurobrucellosis, and endocarditis	Contaminated food and dairy products, occupational contact, and inhalation	World—around 500,000 human cases per year EU—310 human cases in 2019 USA—80–120 cases annually	[5] [6] [16]
Tuberculosis	*Mycobacterium bovis* *M. caprae*	Generalized symptoms (fever, fatigue, arthralgia, and muscle pain), respiratory and cardiac complications, hepatitis, osteoarthritis, central nervous system dysfunction, and orchitis/epididymitis	Inhalation of aerosol, infected milk, dairy products, and meat	EU—147 human cases in 2019 USA—7174 human cases in 2020	[5] [17] [18]
Trichinellosis	*Trichinella* sp.	Diarrhea, abdominal pain at first, fever, myalgia, myocarditis, facial oedemas, and encephalitis	Ingestion of raw or undercooked muscle tissue containing encysted larvae	EU—96 human cases in 2019 USA—90 human cases during 2008–2012	[5] [6] [19]
Yersiniosis	*Yersinia enterocolitica*, *Y. pseudotuberculosis*	Fever, vomiting, abdominal pain, and bloody diarrhea	Eating raw or undercooked pork; ingestion of dairy products, seafood, and vegetables; or drinking contaminated water	EU—6961 human cases USA—nearly 117,000 illnesses per year	[5] [6] [20]
Swine influenza	Swine influenza virus (SIV)	Sneezing, coughing, difficult breathing, fever, lethargy, and decreased appetite	Contact with respiratory discharges or inhalation of exhalated aerosol by sick pig	No specific epidemiological data available, spread worldwide	[21] [22] [23]
Salmonellosis	*Salmonella* sp.	Acute enterocolitis accompanied by inflammatory diarrhea, abdominal pain, fever, nausea, and vomiting	Ingestion of uncooked contaminated foods (eggs, milk, and meat), drinking contaminated water, direct contact with infected animals, their feces and environment, and human-to-human transmission through fecal–oral route	EU—87,923 human cases in 2019 USA—about 1.35 million human illnesses per year Sub-Saharan Africa—535,500 cases of non-typhoidal salmonellosis in 2019	[5] [6] [24] [25]

## Data Availability

Not applicable.
